# Programming the Optoelectronic Properties of Atomically Precise Gold Nanoclusters Using the Conformational Landscape of Intrinsically Disordered Proteins

**DOI:** 10.1002/chem.202502991

**Published:** 2026-01-24

**Authors:** Santiago Rodriguez, Sylvain Kumanski, Zeineb Ayed, Aurélie Fournet, Charlène Bouanchaud, Amin Sagar, Frédéric Allemand, Vladimir A. Baulin, Ute Resch‐Genger, Juan Cortés, Nathalie Sibille, Fabien Chirot, Karl David Wegner, Rodolphe Antoine, Xavier Le Guével, Pau Bernadó

**Affiliations:** ^1^ Centre De Biologie Structurale, Université de Montpellier INSERM, CNRS Montpellier France; ^2^ Université Grenoble Alpes, Institut pour L'avancée Des Biosciences (IAB), Cancer Targets & Experimental Therapeutics INSERM U1209, CNRS UMR 5309 Grenoble France; ^3^ Université Claude Bernard Lyon1‐CNRS, Institut Lumière Matière, UMR5306 Université De Lyon Villeurbanne France; ^4^ BicycleTx Ltd., Cambridge CB21 6GS, United Kingdom. Cambridge UK; ^5^ Departament de Química Física Inorgànica Universitat Rovira i Virgili Tarragona Spain; ^6^ Division Biophotonics Federal Institute for Materials Research and Testing (BAM) Berlin Germany; ^7^ LAAS‐CNRS Université De Toulouse Toulouse France

**Keywords:** gold nanoclusters, intrinsically disordered proteins, NIR‐II, photoluminescence, SAXS

## Abstract

The rational design of hybrid nanomaterials with precisely controlled properties remains a central challenge in materials science. While atomically precise gold nanoclusters (Au‐NCs) offer molecule‐like control over a metallic core, tuning their optoelectronic behavior via surface engineering is often empirically driven. Here, we establish a design principle by demonstrating that the conformational landscape of intrinsically disordered proteins (IDP) can be used as a programmable scaffold to rationally modulate the photophysical properties of a covalently bound Au‐NC. We synthesized a series of bioconjugates between Au_25_ nanoclusters and bioengineered IDPs containing a variable number of cysteine anchoring points. A combination of mass spectrometry, small‐angle X‐ray scattering, and modeling on the conjugates indicates that increasing the number of covalent anchors systematically restricts the conformational ensemble, inducing a progressively more compact protein shell around nanoclusters. This structural rigidification at the interface directly translates into a 15‐fold enhancement of the Au‐NC near‐infrared photoluminescence and a six‐fold increase in its average lifetime. Our findings demonstrate that the conformational plasticity of IDPs and the capacity to engineer them can be harnessed as a molecular tuning knob, moving to a new regime of programmable soft‐matter control over the properties of quantum‐confined nanomaterials for tailored biotechnological applications.

## 1 Introduction

The convergence of nanotechnology and molecular biology has catalyzed the development of hybrid materials where the distinct properties of inorganic nanoparticles and biological macromolecules are synergistically integrated [1, 2]. Among these, atomically precise metal nanoclusters, particularly gold nanoclusters (Au‐NCs), have emerged as exceptional building blocks due to their discrete, molecule‐like electronic structures and size‐dependent photoluminescence (PL) [3, 4, 5, 6]. Conjugating these Au‐NCs to proteins offers a powerful strategy to create functional biomaterials, combining the unique optical and catalytic properties of the metal core with the biocompatibility and specific recognition capabilities of the protein [7, 8].

A critical determinant of the final properties of these nanobioconjugates is the nature of the interface between the inorganic core and the protein shell and its role in tuning the characteristics of nanoclusters [9]. While significant progress has been made, achieving rational, bottom‐up control over this interface to program the emergent properties of the hybrid material remains a formidable challenge. Most studies have employed globular proteins, such as Bovine Serum Albumin (BSA), as templates or carriers [6]. For instance, recent experimental studies, complemented with computational methods of Au‐NCs covalently or electrostatically bound to the BSA, have provided some hints on the relevance of the interface [10, 11]. In another study, the electrostatic interactions between red‐emitting Au‐NCs and protein Tau, a cytosolic Intrinsically Disordered Protein (IDP) associated with Alzheimer's disease, provided multiple analytical and spectroscopic hints for the formation of multimolecular assemblies at the nanobiointerface [12]. These studies suggest that key parameters, such as protein: Au‐NC stoichiometry, binding nature, and environment, strongly influence the optical and electronic properties of these nanobioconjugates. While effective, the fixed tertiary structure of these proteins offers limited tunability over the final conjugate architecture and the local environment of the Au‐NC.

A well‐established principle in nanocluster photophysics is that rigidifying the surface ligand shell can suppress non‐radiative decay pathways, thereby enhancing luminescence quantum yield [13, 14, 15]. This has been achieved through crystallization, encapsulation in rigid polymer matrices, or the use of sterically hindered ligands. However, these methods often result in static systems with fixed properties. An intriguing yet underexplored alternative is to use soft, dynamic biomolecules whose conformational properties can be systematically controlled.

IDPs represent a distinct class of polypeptides that lack a stable tertiary structure, instead existing as highly dynamic conformational ensembles [16, 17, 18]. This conformational plasticity is not random but is encoded in their amino acid sequence and is central to their biological function [19, 20, 21]. Indeed, IDPs perform fundamental biological functions that complement those of their globular counterparts, including signaling, homeostasis, cell‐cycle regulation, transcription, and liquid‐liquid phase separation processes. Importantly, their reduced structural constraints make IDPs excellent platforms to engineer versions for tailored biotechnological applications [22, 23]. We hypothesized that this programmable conformational landscape could be harnessed as a novel tool for materials design. Specifically, we hypothesized that by systematically varying the number of covalent anchoring points between an IDP and an Au‐NC, we could precisely control the accessible conformational space of the protein shell, thereby creating a tunable, soft‐matter interface to program the nanocluster's optoelectronic properties.

**FIGURE 1:**
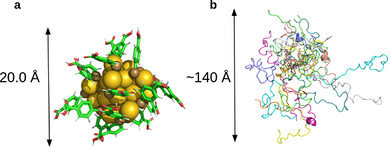
Structural models of the molecules used in this study. (a) Atomistic structure of Au25pMBA18 (pMBA : para‐mercaptobenzoic acid). Gold, carbon, oxygen, and sulphur atoms are displayed in yellow, green, red, and brown, respectively. (b). Ensemble of 20 conformations of the 76‐residue long β2‐WT displayed in different colors to facilitate inspection. The dimension shown (∼140 Å) represents the largest intramolecular distance observed within the pool of conformations.

In this work, we investigate the covalent conjugation of atomically precise Au_25_pMBA_18_ nanoclusters with a series of small (76‐residue long) bioengineered IDPs containing one, two, or three cysteine residues, allowing for a systematic modulation of the number of covalent anchoring points to the Au‐NC surface. Figure 1 displays structural models of both molecules and highlights their different structural properties and their large size disparity, where the protein can adopt conformations almost one order of magnitude larger than the Au‐NC. Through an integrative approach combining mass spectrometry (MS), PL spectroscopy, small‐angle X‐ray scattering (SAXS) complemented and computational modeling, we demonstrate that the number of cysteine anchors acts as a molecular tuning knob. It dictates not only the stoichiometry of the final conjugate but also the degree of compaction of the IDP shell. This programmable structural rigidification at the nano‐bio interface leads to a dramatic and predictable enhancement of the Au‐NC near‐infrared (NIR‐II) PL. Our results establish a new design principle for programmable soft matter–nanoparticle hybrids, where the conformational dynamics of a disordered protein shell define the nano–bio interface and dramatically affect nanoparticle optical properties.

## 2 Results and Discussion

### 2.1 Stoichiometric Control of IDP: Au‐NC Conjugates via Ligand Exchange

We first explored the reaction landscape of the atomically precise Au‐NC, Au_25_pMBA_18_ (pMBA : para‐mercaptobenzoic acid), in the presence of small IDPs. Precisely, we used the 76‐residue long C‐terminal disordered tail of the β2‐adrenergic receptor (β2), which has two free cysteines in its wild type (β2‐2cys), and two single‐point mutations yielding constructs with 1 (β2‐1cys) and 3 (β2‐3cys) free cysteines (see Table S1 in Supporting Information for a complete description of the experimental procedure and characterization). Note that the third cysteine was incorporated in the position of Ser16, a chemically similar amino acid to cysteine, providing an inter‐cysteine distance (22 residues) similar to that of β2‐2cys (27 residues).

When mixing the Au‐NC with each of the three constructs in a ratio 1:2 (protein: Au‐NC) for 3 h, a reduced number of species, which differ from the free Au‐NC and the proteins, could be identified in the native PAGE‐gel in the NIR‐II (1000–1700 nm) and also in the red (667 nm) region, confirming successful conjugation (see Figure S1). In the presence of β2‐2cys, the mixture yielded three bands as revealed by fluorescence and Coomassie staining, indicating that the resulting species contained both the Au‐NC and the protein. Interestingly, an equivalent number of conjugated species was observed when the number of free cysteines of the protein was one or three. In all cases, the two bands closer to the front displayed similar mobility, while the higher ones migrated similarly to the free proteins. It is interesting to note that the positions of these three bands were different depending on the β2 construct used. This suggested that the molecular weight (MW), size, and/or net charge of the bioconjugates changed depending on the number of cysteines.

Then, we explored how the stoichiometry, from 1:0.5 to 1:3, and the reaction times, from 30 min to 24 h, influenced the population of the resulting species (see Figure S2). For the three β2 constructs, the three main bands were present under the vast majority of experimental conditions. However, at high protein: Au‐NC ratios (1:0.5 and 1:1), a fourth band with a migration behavior similar to that of the free protein appeared concomitantly with the decrease in the population of the lowest bands. Interestingly, at the smallest ratios of 1:2 and 1:3 and short reaction times, the highest band was the major species, but its population slowly decreased with time to enhance that of the lowest bands. These observations confirmed that the population of the species formed by mixing the IDP and Au‐NC could be modulated by stoichiometry and reaction time, suggesting their transient nature and complex transformation pathways. However, Au‐NC:IDP mixtures were not strongly influenced by the number of free cysteines, as the three β2 constructs presented the same overall behavior.

**FIGURE 2:**
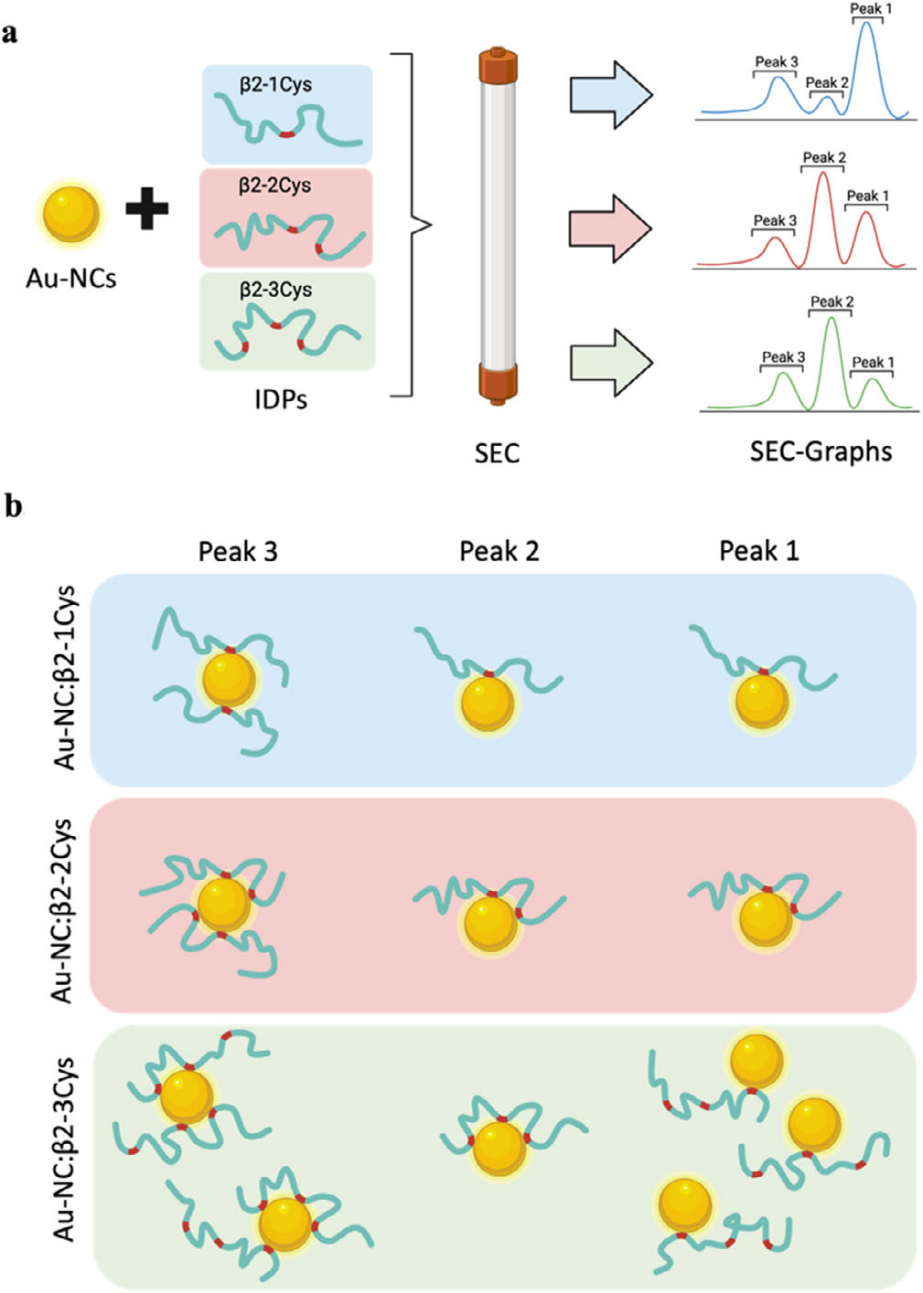
Stoichiometric and structural control of Au‐NC:β2 conjugates. Schematic representation of the protocol followed to synthesize and isolate the species formed upon mixing Au‐NCs with the three constructs of the β2 protein (a), and their conformation suggested by their MS analysis (b). Cysteines within the protein are highlighted in red, while the Au‐NC is represented as a yellow sphere. The formation of intramolecular disulfide bonds is hypothesized for species where not all available cysteines are anchored. When multiple arrangements are compatible with the MS data, several cartoons are displayed.

Next, the nanobioconjugates resulting from the mixture of Au‐NC with the three β2 constructs were chemically, optically, and structurally characterized. To this aim, we performed large‐scale reactions, and the resulting mixtures were subjected to Size‐Exclusion Chromatography (SEC) (Figure 2a). In line with the PAGE gels, three peaks were observed for each of the β2 constructs (see Figure S3). While the most compact species (Peak 1) was the major product for the mixture of Au‐NC with β2‐1cys, the major species for β2‐2cys and β2‐3cys mixtures corresponded to the middle peak (Peak 2). The three peaks resulting from the three constructs were collected, concentrated, and subjected to HPLC‐coupled to MS analysis to identify their chemical composition (see Figure S4). All the experimentally derived MWs could be explained as a single Au_25_ cluster with a defined number of proteins and pMBA molecules (see Table S2). The major species (Peak 1) of the Au‐NC:β2‐1cys mixture showed a MW that was in excellent agreement with a single protein attached to an Au‐NC replacing a single pMBA molecule. Interestingly, when analyzing the MWs of the major species (Peak 2) for the mixtures of Au‐NC with β2‐2cys and β2‐3cys, the values derived were compatible with an Au‐NC:β2 conjugate in which two and three pMBA molecules had been removed, respectively. The correlation between the number of free cysteines of the β2 mutants and eliminated pMBA molecules strongly suggested that the formation of these conjugates followed a substitution mechanism in which the reduced thiols and pMBA molecules were replaced by cysteines. As a consequence, for the β2‐2cys and β2‐3cys conjugates, the protein had multiple anchoring points to Au‐NCs, exposing long flexible loops and tails to the solvent (Figure 2b). To better understand the pMBA/cysteine substitution mechanism, we mixed the Au‐NC with an engineered β2 construct without cysteines (β2‐0cys, Table S1). Interestingly, the only band appearing in the PAGE‐gel, as revealed by Coomassie staining, corresponded to that of the protein, indicating that no conjugates were formed when no free cysteines were present in the protein (see Figure S5).

MS data obtained for the minor peaks provided information on the versatility of the Au‐NCs to interact with the proteins. In terms of stoichiometry, Peak 2 for Au‐NC:β2‐1cys and Peak 1 for Au‐NC:β2‐2cys were indistinguishable from the corresponding major peaks. Interestingly, the MW derived from Peak 1 of Au‐NC:β2‐3cys indicated that a single pMBA had been removed from the Au‐NC, suggesting that this species presented two free cysteines in solution. Given the reactivity of cysteine thiols with Au‐NCs, we speculate that the formation of an intramolecular disulfide bond could be responsible for this observation. The MWs derived from the three species with the shortest elution time in the SEC (Peak 3) showed the presence of species with a 1:2 stoichiometry, indicating that these Au‐NCs could simultaneously bind two protein chains. Interestingly, the conjugates obtained from β2‐1cys and β2‐2cys had all cysteines of both proteins engaged in binding to the Au‐NC, implying that up to four pMBA molecules could be removed from the Au‐NC (Figure 2). A slightly different scenario occurred for Peak 3 of the β2‐3cys conjugate. Although two proteins form a complex with the Au‐NC, only four out of the six available cysteines were engaged in Au‐NC binding. Again, the presence of intra‐ or intermolecular disulfide bridges, hampering the interaction with the gold atoms at the Au‐NC surface, could not be discarded.

The ensemble of these results confirms that the number of cysteine residues in the IDP sequence provides a programmable handle to precisely control the stoichiometry and covalent connectivity of the final nanobioconjugate.

### 2.2 Programmed Photoluminescence Enhancement by Cysteine Anchoring

Having established stoichiometric control, we investigated how the number of anchoring points modulates the optical properties of the Au‐NC. The absorbance spectra of the Au‐NCs dispersed in PBS buffer exhibited characteristic bands and shoulders at 450, 480, 690, and 800 nm, which were attributed to molecular‐like transitions occurring within the core (Au_13_ kernel) and shell (Au_n_R_m_ staple motifs) of the Au_25_ nanocluster [24, 25]. The complexation of this Au‐NC with the three β2 constructs through their cysteines seemed to moderately alter the icosahedral structure of the Au‐NC, as confirmed by the slight blue‐shift of the band at 690 nm (see Figure S6) [25]. The resulting absorption spectra were smoother than those of the Au‐NC, and some of the typical bands of the nanoparticles disappeared, which is due to the low concentration of Au‐NC and the increasing influence of scattering. Note that, in the absence of Au‐NCs, the different β2 constructs exhibited the typical protein absorption profiles, with a band at 280 nm arising from aromatic amino acids (data not shown).

**FIGURE 3:**
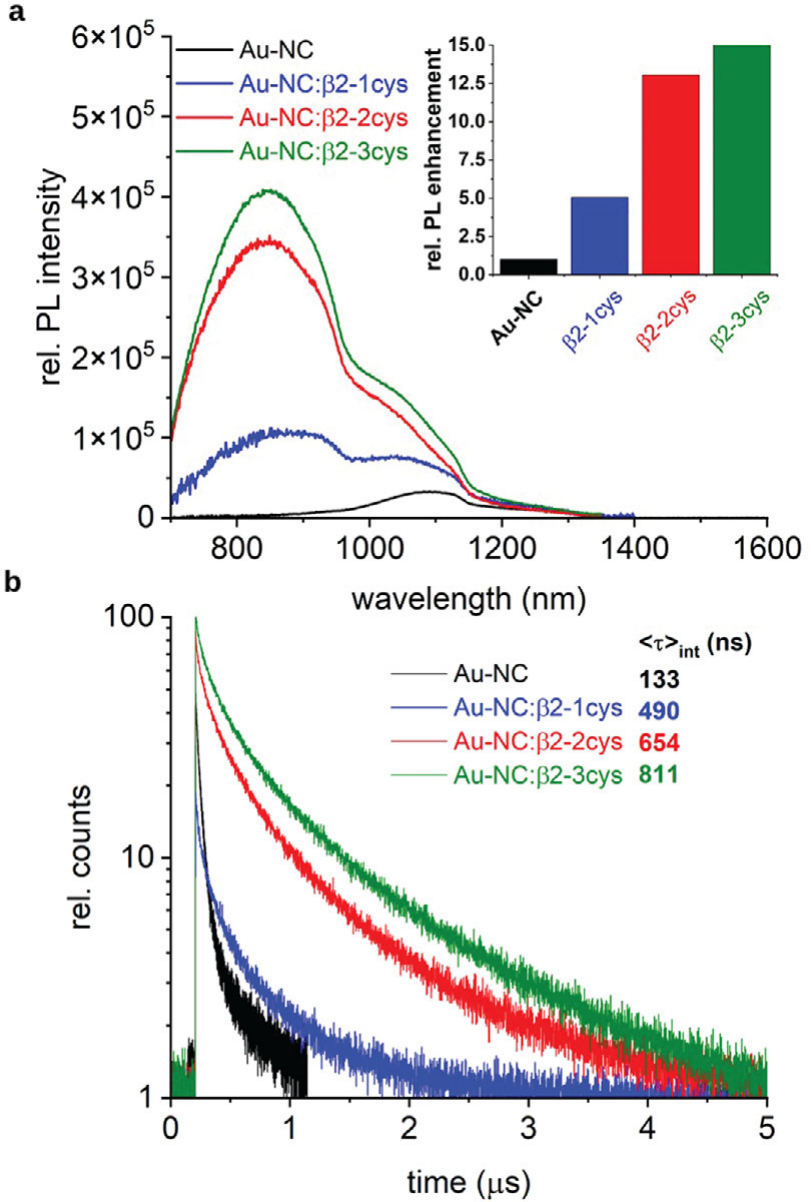
Programmable photoluminescence enhancement. (a) Absorbance‐corrected PL emission spectra of the major species of the β2 conjugates (Peak 1 for β2‐1cys, and Peak 2 for β2‐2cys and β2‐3cys). Excitation 𝛌exc= 450 nm. The inset quantifies the PL enhancement relative to the free Au‐NC. (b) PL decay curves of the three major Au‐NC:β2 species and the calculated intensity‐weighted average PL lifetimes. Excitation was at 𝛌exc= 510 nm. The intensity‐weighted average lifetimes (< τ >int) show a systematic increase with the number of cysteine anchors.

PL spectra of Au‐NCs dispersed in PBS buffer typically exhibit a relatively broad emission band peaking at 1100 nm with a shoulder at 1250 nm. While the exact origin of the NIR‐II PL remains unclear, recent studies indicate that the ligand plays a crucial role in modulating the NIR‐II PL through intramolecular charge transfer (ICT) [26, 27]. We observed a striking enhancement of the Au‐NC PL upon protein conjugation, with the number of cysteines directly influencing the amplification of a shorter‐wavelength PL emission at 850 nm (Figures 3a and S6). Notably, the PL intensity increased more than 15‐fold for the complex containing three cysteines, with a significant 3‐fold increase for two cysteines compared to one cysteine. This enhancement appeared to be wavelength‐independent (see Figure S6). These observations demonstrate that the number of covalent tethers between the IDP and the Au‐NC acts as a rational tuning knob for the conjugate photophysical output.

To elucidate the mechanism behind this enhancement, we performed time‐resolved measurements providing insight into the PL decay kinetics, which turned out to be complex (Figure 3b). Fitting of the decay curves to a three‐component exponential model yielded an intensity‐weighted average PL lifetime (< *τ* >_int_) of 133 ns for isolated Au‐NCs. Upon conjugation, we observed a systematic and significant increase in the average PL lifetime, which scaled directly with the number of cysteine anchors: 490 ns for the 1‐cys conjugate, 654 ns for the 2‐cys conjugate, and 811 ns for the 3‐cys conjugate (Figures 3b andS6).

While a previous study showed a moderate impact on the PL when replacing pMBA with cysteine on atomically precise Au_25_ nanoclusters [10], our notable PL enhancement suggests a pivotal role of the interface between the disordered protein and the Au‐NC. Indeed, the simultaneous increase in both PL intensity and lifetime is a hallmark of the suppression of non‐radiative decay processes [28]. This indicates that the progressively constrained IDP shell, enforced by multiple anchoring points, creates a more rigid and protective microenvironment at the Au‐NC surface, thereby inhibiting vibrational quenching mechanisms and favoring radiative recombination. It should be highlighted that PL enhancement has also been observed for Au‐NCs non‐covalently bound to IDPs [12] (up to 20‐fold at 600 nm) or to globular proteins such as BSA [10, 11] (up to 4.5‐fold at 1065 nm). Those studies, supported by isothermal titration calorimetry (ITC) experiments and circular dichroism for IDP and by molecular dynamic simulations in the case of BSA, demonstrate the role of rigidity and reduced polarity at the Au‐NCs‐protein interface in suppressing non‐radiative pathways leading to PL enhancement [29]. However, PL changes in such bioconjugates should be interpreted with caution. Protein conformational modifications driven by solvent and temperature in biological environments can occur, facilitating intercluster hydrogen bonding, thereby significantly affecting the optical properties, including PL spectra and intensity [30, 31, 32, 33].

### 2.3 SAXS Reveals Anchor‐Dependent Compaction of the IDP Shell

**FIGURE 4:**
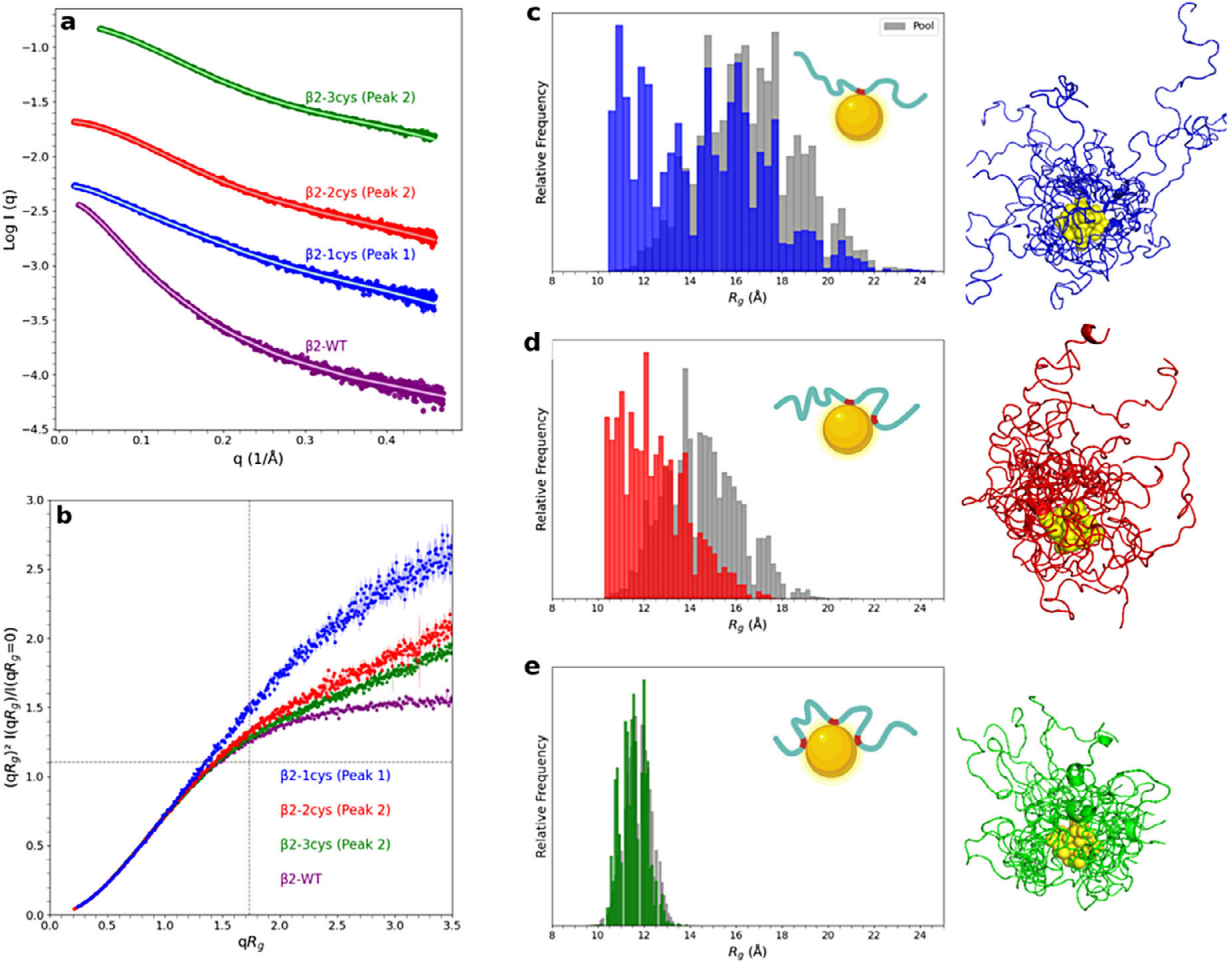
SAXS analysis of anchor‐dependent IDP compaction. (a) SAXS curves for the Au‐NC containing β2‐1cys, β2‐2cys, β2‐3cys major species, and β2‐WT, which encompasses two free cysteines. (b) Dimensionless Kratky plots for the β2‐1cys, β2‐2cys, β2‐3cys main peaks and β2‐WT. (c‐e) Rg distributions for the major 1:1 conjugate species from the initial pool of conformations (grey) and the final EOM‐selected ensemble (red) for (c) β2‐1cys (blue), (d) β2‐2cys (red), and (e) β2‐3cys (green), with their corresponding ensemble. The increasing number of anchors progressively restricts the IDP to more compact conformations.

The structural characterization of the Au‐NC conjugates was performed by SAXS, a biophysical technique that is extremely sensitive to the size and shape of nano‐objects in solution [34, 35]. Batch or the SEC coupled to SAXS (SEC‐SAXS) measuring modes were used depending on the amount of conjugate available (Figures 4a and S7). For the three β2 constructs, the curves obtained for Peaks 1 and 2 indicated that these species were very compact, with radii of gyration (*R_g_*) between 11.2 and 14.0 Å (see Table S2). Note that β2, in the absence of Au‐NCs, has a *R_g_* of 24.3 ± 0.1 Å (Figure 4) [36]. This strong reduction in the *R_g_* was attributed to the presence of small nanoparticles with a diameter of ∼20 Å with a MW (7.7 kDa) that is similar to that of the protein (8.1 kDa). Note that *R_g_* defines the particle mass distribution around its center of mass (CoM). Therefore, the incorporation of the Au‐NCs approaches the CoM to the cluster, strongly modifying the resulting *R_g_* of the conjugates and their SAXS curve. Interestingly, when analyzing the dimensions of the major species for the three constructs, where all cysteines were engaged in Au‐NC binding, a systematic reduction of the *R_g_* was observed when increasing the number of cysteines (14.0 ± 0.1, 12.2 ± 0.1, and 11.3 ± 0.1 Å for β2‐1cys, β2‐2cys, and β2‐3cys complexes, respectively). Given that the inorganic Au25 core is identical in all conjugates, this pronounced compaction must arise from a restriction in the conformational space sampled by the IDP shell. In agreement with the simultaneous complexation to two proteins, larger sizes were observed for the species corresponding to Peak 3. Again, a compaction was observed when passing from β2‐1cys to β2‐2cys conjugates (with *R_g_* s of 17.1 ± 0.2 Å and 14.9 ± 0.1 Å, respectively) due to the larger number of cysteines bound to the Au‐NCs. A different scenario was observed for Peak 3 of the Au‐NC:β2‐3cys, which has a *R_g_* closer to the β2‐1cys (17.5 ± 0.1 Å), probably due to the presence of β2 chains engaging a single cysteine in the complex (Figure 2). Despite the compact structure of the conjugates, the absence of a maximum in the Kratky representations of all measured SAXS curves indicated that the protein chains displayed an important degree of mobility upon binding to the Au‐NC (Figures 4b S8). Interestingly, the slope of the Kratky plots at large angles, which reports on the compactness of the sample, was in line with the number of cysteines of the β2 construct and the *R_g_* values [37, 38, 39].

We then used the SAXS data to derive detailed structural models for the different conjugates to analyze the conformational behavior of β2 when bound to an Au‐NC. Therefore, we first built atomic models of all assemblies using the MS information regarding the number of cysteines engaged in the complex (Figure 2b). While the coordinates of the Au_25_pMBA_18_ were considered fixed, polypeptide chains were built using sampling methods implemented in the MoMA software suite [40], placing the bound cysteines at the position of randomly chosen pMBA molecules of the Au‐NC (see Supporting Information for details). These pools were then used to fit the experimental SAXS data with the Ensemble Optimization Method (EOM) [41, 42], which selects a sub‐ensemble of conformations that best represents the species in solution (Figures 4a and S7, and Table S2). An excellent description of all SAXS profiles was obtained, with 𝝌^2^ values below 1.14, indicating that the resulting ensembles were a good representation of the different Au‐NC complexes in solution. The comparison between the *R_g_* distributions from the initial pool of conformations and those obtained from the EOM fit is an excellent tool to quantitatively evaluate the compactness of protein ensembles in solution (Figures 4 and S9) [43, 44]. The EOM analyses of the SAXS curves of these major species indicated that the conformational space covered by these conjugates strongly depended on the number of cysteines present in the construct (Figure 4c–e). The β2‐1cys conjugate, which is bound to the Au‐NC through a single cysteine, revealed a broad range of *R_g_* values, although the population of very compact conformations, with *R_g_* values smaller than 15 Å, was enormously enriched with respect to those available in the conformational pool. A similar conformational enrichment occurred for the β2‐2cys conjugate, even though the available conformational space available for this species was notably reduced due to the double anchoring point. Finally, the conformational space of the β2‐3cys conjugate was very narrow, with *R_g_* values from 10 to 14 Å, and very similar to that observed for the available conformations with the three cysteines bound to the Au‐NC. These observations suggest that, in addition to the cysteine anchoring, other interactions occur at the interface between the protein and the Au‐NC that induce a general increase in particle compactness (Figure 4c–e). To support this observation, we compared the *R_g_* distributions of the ensemble derived from the EOM fitting for Peak 2 of the Au‐NC:β2‐2cys with that of the same ensemble after synthetically removing the Au‐NC (see Figure S10). The systematic shift toward smaller *R_g_* values probed the effect of the presence of a heavy Au‐NC. When comparing these distributions with the EOM result of β2‐WT, which is much more extended, we noticed that the main species of Au‐NC:β2‐2cys adopted very compact conformations, necessarily originating from the non‐covalent interactions of the protein with the Au‐NC surface (see Figure S10).

In line with the observations for the major species, the enrichment in very compact IDP conformations was also observed for the minor peaks of the conjugates (Peak 1 of β2‐2cys and β2‐3cys, and Peak 2 of β2‐1cys), suggesting similar interaction mechanisms at the surface of the Au‐NC (see Figure S9). A different scenario emerged when analyzing the data corresponding to Peak 3 for the three β2 constructs, which contain two disordered chains per Au‐NC. In this case, the selected conformations displayed size distributions that were very similar to those of the original pools (see Figure S9). We speculate that the steric hindrance hampered both proteins to simultaneously experiencing extensive non‐covalent interaction at the Au‐NC interface, as occurred with the species containing a single disordered chain.

The SAXS‐driven structural modelling demonstrates that the IDP shell is not merely a passive coating but a programmable soft material whose ensemble properties can be rationally tuned by changing the number of anchoring points and the electrostatic nature of the protein.

## 3 Conclusions

In this study, we have demonstrated the design of programmable nanobiomaterials for atomically precise Au‐NC by harnessing the unique conformational properties of IDPs. By systematically varying the number of cysteine residues in an engineered IDP, we achieved precise stoichiometric control over its covalent conjugation to an atomically precise Au‐NC via a ligand exchange mechanism. The observation of these findings for a large Au‐NC containing 102 Au atoms supports the assumption of the general applicability of this design approach (see Figure S11).

This molecular‐level control over the covalent connectivity at the nano‐bio interface serves as a powerful tool to program the material's emergent properties. Our integrative structural and optical characterization demonstrates a clear causal chain: increasing the number of anchoring points progressively restricts the conformational ensemble of the IDP shell, forcing it into a more compact and rigid state. This programmed rigidification of the soft protein corona directly suppresses non‐radiative decay pathways at the nanocluster surface, resulting in a dramatic, predictable enhancement of its NIR‐II PL and a six‐fold extension of its excited‐state lifetime.

While alternative strategies to enhance PL properties of Au‐NCs have been explored with peptide engineering, IDPs offer additional possibilities to be engineered, enriching the capacity to tune the optical properties of Au‐NCs [45, 46, 47]. IDPs, with lesser structural constraints than their globular counterparts, enable deeper exploration of the sequences. Moreover, the capacity to produce them recombinantly allows the design of chimeras, to fuse them with other proteins harnessing distinct (complementary) functions, or to incorporate signal peptides to target cellular organelles. Importantly, in the context of the Au‐NC functionalization, the introduction of multiple cysteines along the sequence is not expected to change the conformational properties if the formation of intramolecular disulfide bonds is avoided. The number of cysteines, as well as the length and composition of the inter‐cysteine stretches, are relevant parameters that will strongly modify the resulting conjugates and their properties. Indeed, the length and solvent exposure of the loops emerging upon anchoring two consecutive cysteines to the cluster will depend on the designed sequence. However, a minimal distance between consecutive cysteines enabling the simultaneous anchoring must be considered. Although we have not explored this parameter, our simulations suggest that four residues should be enough to obtain two‐site binding, a number that could differ depending on the size of the Au‐NC. Importantly, according to our results, all these design possibilities can be systematically investigated using our modelling approaches and validated by SAXS. However, recent advances in computational modeling of Au‐NCs [48, 49] would allow a more detailed description of these conjugates, accounting for the steric hindrance imposed by the non‐exchanged ligands, the transient interaction with other amino acids such as methionine, and the entropic penalty resulting from binding multiple cysteines.

Overall, this study goes beyond the traditional view of proteins as simple targeting moieties or stabilizing agents in nanomedicine. It reframes IDPs as a class of programmable soft matter, whose encoded conformational dynamics can be rationally engineered to tune the fundamental physicochemical properties of an inorganic nanomaterial. The ability to program the structure and optoelectronic output of these hybrid materials through simple sequence modifications opens new avenues for the bottom‐up design of advanced functional materials. This level of control is poised to enable the creation of next‐generation nanoprobes with tailored optical responses for applications in selective biosensing, multiplexed bioimaging, and as platforms for investigating the fundamental physics of soft‐hard matter interfaces.

## Conflicts of Interest

The authors declare no conflict of interest.

## Supporting information




**Supporting File 1**: Detailed experimental methods and characterization of all products, along with analytical data, are given in the Supporting Information. Moreover, figures with additional data on sample purification, as well as optical and structural analyses are also provided in the supporting information. The authors have cited additional references within the Supporting Information [50, 51, 52, 53, 54, 55].
