# Supplementary material for: Programming the Optoelectronic Properties of Atomically Precise Gold Nanoclusters Using the Conformational Landscape of Intrinsically Disordered Proteins
Source: Chemistry. 2026 Jan 24;32(30):e02991. doi: 10.1002/chem.202502991 (PMC13472344; doi:10.1002/chem.202502991)
Supplement: Supplementary file 1 [file CHEM-32-e02991-s001.pdf]

Supplementary Information

Programming the Optoelectronic Properties of Atomically Precise Gold Nanoclusters using the Conformational Landscape of Intrinsically Disordered Proteins

Santiago Rodriguez[1,#], Sylvain Kumanski[1,#], Zeineb Ayed[2,#], Aurélie Fournet[1], Charlène Bouanchaud[3], Amin Sagar[4], Frédéric Allemand[1], Vladimir A. Baulin[5], Ute Resch-Genger[6], Juan Cortés[7], Nathalie Sibille[1], Fabien Chirot[3], K. David Wegner[6], Rodolphe Antoine[3], Xavier Le Guével[2,], Pau Bernadó[1,*]*

1- Centre de Biologie Structurale, Université de Montpellier, INSERM, CNRS, 29 rue de Navacelles, 34090, Montpellier, France.

2-Université Grenoble Alpes, INSERM U1209, CNRS UMR 5309, Institut pour l'Avancée des Biosciences (IAB), Cancer Targets & Experimental Therapeutics, 38000 GRENOBLE, France.

3- Institut Lumière Matière, UMR5306, Université Claude Bernard Lyon1-CNRS, Université de Lyon, 69622, Villeurbanne Cedex, France.

4- MoleculMind AI. London, UK.

5- Departament de Química Física inorgànica, Universitat Rovira i Virgili, Tarragona 43007, Spain.

6- Federal Institute for Materials Research and Testing (BAM), Division Biophotonics, Richard-Willstaetter-Str. 11, 12489 Berlin, Germany

7- LAAS-CNRS, Université de Toulouse, CNRS, F-31400 Toulouse, France.

# Equivalent contribution

***Corresponding authors:** Xavier Le Guével (xavier.le-guevel@univ-grenoble-alpes.fr) and Pau Bernadó (pau.bernado@cbs.cnrs.fr)

Experimental section

Synthesis and purification of atomically precise Au-NCs

The synthesis of $Au_{25}pMBA_{18}$ followed the protocol by Bertorelle *et al*[1]. First, 79 mg of 4-Mercaptobenzoic acid (pMBA) were dissolved in 40 mL of methanol and 4 mL of tributylamine. Then, 100 mg of tetrachloroauric acid trihydrate were added at room temperature and stirred 30 minutes to form a gold thiolate/tributylamine complex. Then, to induce a slow reduction of gold, 200 mg of trimethylamine borane were added under stirring for 2 h before adding 200 mg more. The solution was left under stirring overnight to achieve the formation of the clusters.

The precipitation of the clusters was induced by adding 1 mL of 10% NH_4OH solution and 30 mL of diethyl ether (Et_2O). After centrifugation, the supernatant, which contains unwanted products, was removed. A second redispersion/precipitation cycle (1 mL H_2O; 5 mL of MeOH and 20 mL of Et_2O) was done. Purification of the $Au_{25}pMBA_{18}$ clusters was done by selective precipitation. The precipitate was redispersed in 5 mL of water and then 700 mg of ammonium acetate were added, followed by 20 mL of MeOH. Under these conditions, a precipitate forms and it is separated by centrifugation. The precipitate was discarded and the supernatant was further precipitated by adding Et_2O. Two cycles of redispersion/precipitation were done to improve purity of the cluster. The pellet was then resuspended in water and containing 100 mM Triethylammonium acetate to achieve a final concentration of 1 mg/mL.

Protein constructs

The C-terminal part of the β2-adrenergic receptor (β2) was cloned into a pET-1a expression vector as described by Guillien et *al*[2]. This plasmid, pETGST-1a-β2AR WT, allowed the

expression of a (His)6-GST-tagged protein. In the wild type sequence, the β2 carries two natural cysteines at the positions 39 and 67. Here, we have engineered different mutants named pETGST-1a-β2AR C39A+C67A, pETGST-1a-β2AR C67A and pETGST-1a-β2AR S16C containing zero, one or three cysteines, respectively (Table S3). All these vectors were constructed using the QuikChange Site-directed mutagenesis kit (Agilent™). pETGST-1a-β2AR C67A and pETGST-1a-β2AR S16C plasmids were first synthesized using pETGST-1a-β2AR WT vector as a template, and then pETGST-1a-β2AR C39A + C67A was constructed using vector pETGST-1a-β2AR C67A as a template.

Protein expression and purification

All the plasmids were transformed in *Escherichia coli* BL21 DE3 strain (Fisher Scientific, Illkrich, France). Proteins were expressed in self-inducible medium ZYM50-52 at 37°C. After harvesting, bacteria were resuspended and lysed by sonication in 20 mM Tris pH 7.5, 300 mM NaCl, 2 mM dithiothreitol (DTT) named Buffer A and supplemented with anti-protease (Complete EDTA free tablet (Roche, Saint Quentin Fallavier, France)). Cell debris were removed by centrifugation, the soluble fraction was loaded into a 5 mL HisTrap Excel column (Cytiva, Uppsala, Sweden, 17371906). The column was washed in Buffer A supplemented with 10 mM imidazole. Proteins were eluted with a linear gradient of Buffer A supplemented with 500 mM imidazole. The eluate was dialysed in Buffer A containing TEV protease (1/50 (w/w) protease/protein) at 4°C overnight. Proteases and His-GST tag were removed using a gravity column containing Glutathione Sepharose 4B (GE Healthcare, Uppsala, Sweden, 17-0756-05) and Complete His-Tag purification resin (Roche, Mannheim, Germany, 05893682001). Proteins were concentrated using a 3 kDa Amicon concentrator (Merck, Cork, Ireland, UFC900324) then injected into a HiLoad 16/600 Superdex 75pg column (GE Healthcare, Uppsala, Sweden, 28-9893-

33) with 50 mM Bis Tris pH 6.7, 50 mM NaCl, 2 mM DTT. The purified proteins were stored at -20°C until use.

Preparation and purification of the Au-NC:β2 complexes

The buffer protein was exchanged to PBS-1X supplemented with 100 µM TCEP (Tris(2-CarboxyEthyl)Phosphine) then diluted to 100 µM. The mixing of proteins with Au-NC at a 1:2 ratio (15 µM : 30 µM) was done at room temperature with low agitation for 3h. The mixture was concentrated to 10X using a 3 kDa Amicon concentrator (Merck, Cork, Ireland, UFC800324) then injected into a Superdex 75 10/300 GL column (GE Healthcare, Uppsala, Sweden, 17-5174-01) equilibrated in PBS-1X. Purified samples were stored at -20°C until use.

Polyacrylamide Gel Electrophoresis (PAGE)

PAGE separation was carried out using a vertical gel electrophoresis unit with a size of 0.2 cm × 20 cm × 20 cm. The separating and stacking gels were prepared from acrylamide monomers with the total contents of 20 and 7 wt %, respectively [40% acrylamide–bis(acrylamide) 29:1]. The eluting buffer is a standard TBE buffer (89 mM Tris, 89 mM boric acid, 2 mM ethylenediaminetetraacetic acid). The Au-NC:β2-2Cys complexes were dissolved in a 15% (v/v) glycerol/water solution. The sample solutions were loaded onto the stacking gel (200 µL) and eluted for 2 h at a constant voltage mode (150 mV) to achieve sufficient separation of the peaks. The gel was then removed from the casket and placed under either a visible-NIR camera with a couple excitation/emission at 647nm/667 nm (Typhoon FLA 9500 (GE Healthcare Life Sciences)) or under a SWIR camera (Nirvana 640-ST coupled with 35 mm lens (Navitar) and long pass filter at 900 nm (Thorlabs) following an excitation with a Laser at $\lambda_{exc.}$= 808 nm.

HPLC-coupled mass spectrometry

Purified samples were concentrated to at least 10X using a 3 kDa Amicon concentrator (Merck, Cork, Ireland, UFC800324) before the Mass spectrometry (MS) analyses. MS was performed at the OLAF Mass spectroscopy platform of the University of Montpellier with a Synapt G2-S (Waters, UEB205) equipped with an electrospray ionization source. The spectrometer was connected to a UPLC Acquity H-Class (Waters) equipped with a Bioresolve RP mAb 150x2.1mm, 1.7 µm (Waters) column and the UV chromatogram was recorded at 280 nm. Data were recorded using positive and negative ionization modes, although only results obtained using the positive mode are reported.

Optical and spectroscopic characterization of complexes

All samples were diluted in PBS buffer (10mM) and PL measurements were performed at 0.1 OD at λ = 450 nm for steady state measurement and λ = 510 nm for time-resolved measurements. Absorbance measurements were performed using a Cary5000 (Agilent) UV-Vis-NIR spectrophotometer in the range of 300-1100 nm. Steady-state photoluminescence spectra were measured from 700–1600 nm with a calibrated FSP 920 (Edinburgh Instruments, Edinburgh, United Kingdom) spectrofluorometer equipped with a nitrogen-cooled PMT R5509P. Time-resolved measurements were performed in the wavelength region of 900 - 940 nm using a FLS 920 (Edinburgh Instruments, Edinburgh, United Kingdom) lifetime spectrofluorometer equipped with an EPL-510 (Edinburgh Instruments, Edinburgh, United Kingdom) picosecond pulsed diode laser (excitation wavelength of 510±10 nm; power of 5 mW) and a fast PMT R2658P from Hamamatsu, respectively. The PL decays were fitted with a multi-exponential model, see Eq. (1)

$$I(t) = \sum_{i}^{n} A_i \; e^{\left(\frac{-t}{\tau_i}\right)} \qquad (1)$$

where A_i are the amplitudes and τ the lifetimes of the respective decaying species. These data were calculated with a reconvolution fitting procedure using the FLS-920 spectrometer software. The fit quality was evaluated from the corresponding χ^2 values. The average lifetimes were calculated as an intensity-weighted average, see Eq. (2)

$$< \tau >_{int} = \frac{\sum_i^n A_i \tau_i^2}{\sum_i^n A_i \tau_i} \qquad (2)$$

where, τ_i are the lifetimes of each component and A_i the corresponding relative fractional amplitudes.

Small-angle X-ray scattering measurement and raw data analyses

Small-angle X-ray scattering (SAXS) data were acquired for the different purified peaks for the Au-NC:β2 conjugates at the SWING beamline at the SOLEIL Synchrotron (Saint-Aubin, France) using X-ray wavelength of 1.03 Å and a sample-to-detector distance of 2.00 m[3]. Samples were measured at 15°C either in batch or in Size-Exclusion Chromatography coupled to SAXS (SEC-SAXS) modes in PBS-1X. For the SEC-SAXS mode, 45 µL of samples were injected into a 3 mL Superdex 75 5/150 GL column (Cytiva, Uppsala, Sweden) pre-equilibrated into the same buffer and were eluted at 0.2 mL/min.

The scattering profiles measured covered a momentum transfer range of 0.002 $Å^{-1} < q < 0.5$ $Å^{-1}$. In SEC-SAXS experiments, the scattering patterns of the buffer were recorded before the void volume of the column (1 mL). SAXS data were processed using CHROMIX to automatically select

frames corresponding to buffer and sample, perform buffer subtraction and average profiles[4]. The averaged SAXS curves were analyzed using Primus from ATSAS software package[5].

SAXS-driven modelling of the gold-conjugated nanostructures

The modeling of each of the Au-NC:β2 complexes was performed using the conformational sampling algorithms implemented in the MoMA software suite (https://moma.laas.fr/)[6]. For species with a single cysteine engaged in the interaction (Peak 1 and Peak 2 of Au-NC:β2-1Cys, and Peak 1 of Au-NC:β2-3Cys), the building procedure was the following: (*i*) A single extended conformation of β2 was collected and an Au-NC particle with 17 pMBA molecules was manually approached with PyMOL Molecular Graphics System 3.0 (Schrödinger, LL) to the free cysteine, positioning the free gold atom (after removing the pMBA) at 2.2–2.6 Å to the sulfur atom[7]. (*ii*) From this initial state, a conformational ensemble (20,000 conformations) of β2 was built with MoMA by fixing the position of the bound cysteine. (*iii*) The sampled conformations were submitted to a filtering procedure in order to select those sterically compatible with the Au-NC[6]. Concretely, protein conformations with at least one atom within 1 Å of any gold atom were discarded, unless that atom was in the flanking region (±3 residues) of the bound cysteine. A similar procedure was used to build species with 2 or 3 anchored cysteines and with two different chains. The main difference was the initial β2 conformations, which were built by forcing MoMA to position the anchored cysteines in close proximity to make chemically compatible interactions with the Au-NC after removing the specified number of pMBA molecules. In all the cases, the filtering criteria were maintained.

After filtering, the SAXS profile for each conformation of each conjugate was calculated using CRYSOL[8], setting the number of spherical harmonics to 35 and treating hydrogen atoms

explicitly. The calculated SAXS profiles were fitted to experimental data using GAJOE from the EOM suite, running 500 cycles of 2,000 generations each, with an ensemble size of 10, disallowing repetitions[9,10].

REFERENCES

1. Bertorelle F, Russier-Antoine I, Comby-Zerbino C, Chirot F, Dugourd P, Brevet PF, et al. Isomeric Effect of Mercaptobenzoic Acids on the Synthesis, Stability, and Optical Properties of Au25(MBA)18 Nanoclusters. ACS Omega. 2018;3: 15635–15642. doi:10.1021/acsomega.8b02615

2. Guillien M, Mouhand A, Fournet A, Gontier A, Navia AM, Cordeiro TN, et al. Structural Insights into the Intrinsically Disordered GPCR C-Terminal Region, Major Actor in Arrestin-GPCR Interaction. Biomolecules. 2022;12: 617. doi:10.3390/biom12050617

3. Thureau A, Roblin P, Pérez J. BioSAXS on the SWING beamline at Synchrotron SOLEIL. J Appl Crystallogr. 2021;54: 1698–1710. doi:10.1107/S1600576721008736

4. Panjkovich A, Svergun DI. CHROMIXS: Automatic and interactive analysis of chromatography-coupled small-angle X-ray scattering data. Bioinformatics. 2018;34: 1944–1946. doi:10.1093/bioinformatics/btx846

5. Manalastas-Cantos K, Konarev P V., Hajizadeh NR, Kikhney AG, Petoukhov M V., Molodenskiy DS, et al. ATSAS 3.0: Expanded functionality and new tools for small-angle scattering data analysis. J Appl Crystallogr. 2021;54: 343–355. doi:10.1107/S1600576720013412

6. Estaña A, Sibille N, Delaforge E, Vaisset M, Cortés J, Bernadó P. Realistic Ensemble Models of Intrinsically Disordered Proteins Using a Structure-Encoding Coil Database. Structure. 2019;27: 381-391.e2. doi:10.1016/j.str.2018.10.016

7. Jadzinsky PD, Calero G, Ackerson CJ, Bushnell DA, Kornberg RD. Structure of a thiol monolayer-protected gold nanoparticle at 1.1 Å resolution. Science (1979). 2007;318: 430–433. doi:10.1126/science.1148624

8. Franke D, Petoukhov M V, Konarev P V, Panjkovich A, Tuukkanen A, Mertens HDT, et al. ATSAS 2.8: a comprehensive data analysis suite for small-angle scattering from macromolecular solutions. J Appl Crystallogr. 2017;50: 1212–1225. doi:10.1107/S1600576717007786

9. Tria G, Mertens HDT, Kachala M, Svergun DI. Advanced ensemble modelling of flexible macromolecules using X-ray solution scattering. IUCrJ. 2015;2: 207–17. doi:10.1107/S205225251500202X

10. Bernadó P, Mylonas E, Petoukhov M V., Blackledge M, Svergun DI. Structural characterization of flexible proteins using small-angle X-ray scattering. J Am Chem Soc. 2007;129: 5656–5664. doi:10.1021/ja069124n

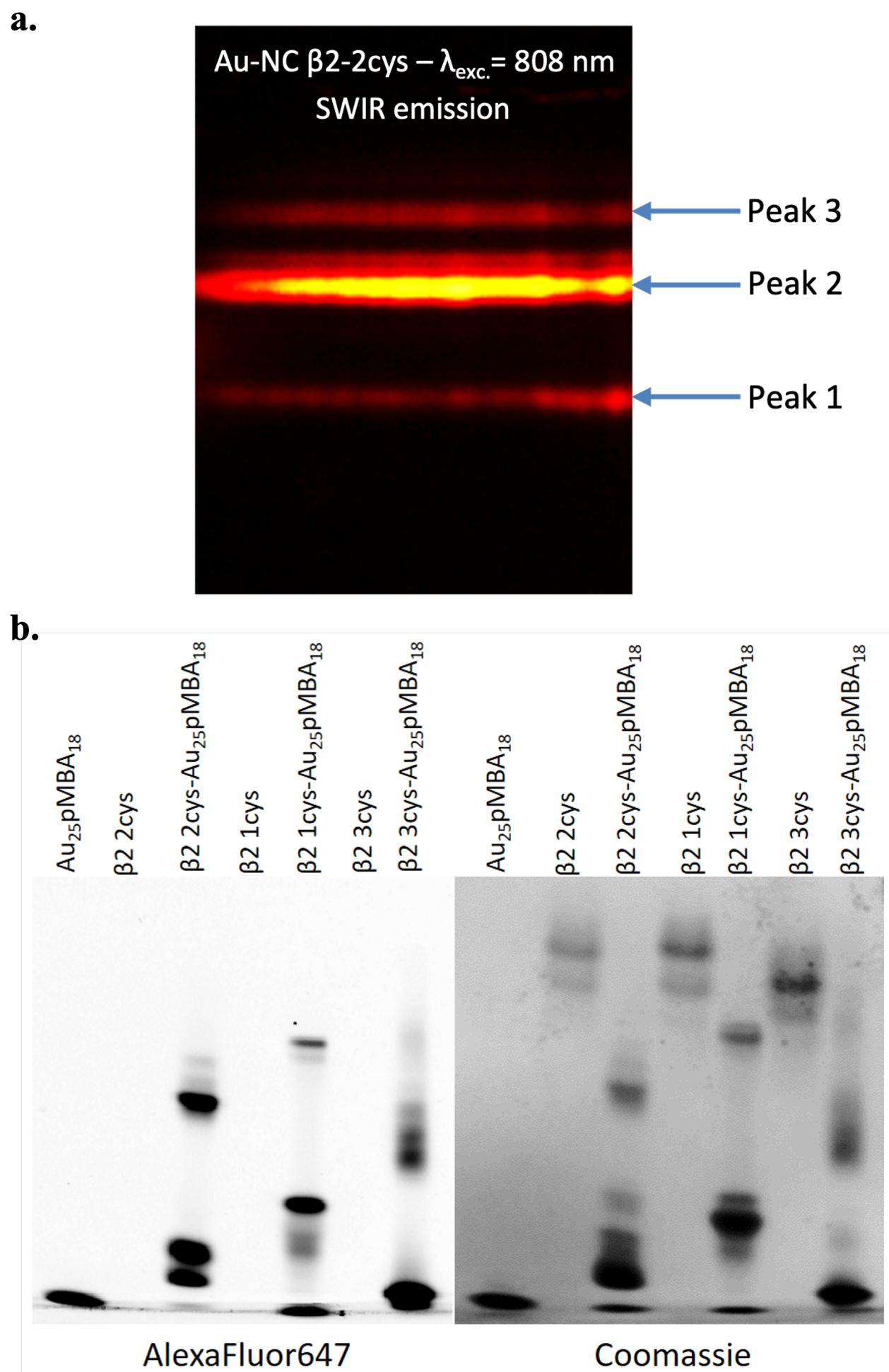

Figure S1. (a) Photoluminescent image of PAGE migration profile of Au-NC: β2-2cys complexes in a native gel in the SWIR following an excitation at $\lambda_{exc.}$= 808 nm. **(b)** Native PAGE-gel revealed by fluorescence emission at 667 nm (left) and Coomassie (right) of the mixtures of the $Au_{25}pMBA_{18}$ with the 3 β2 constructs.

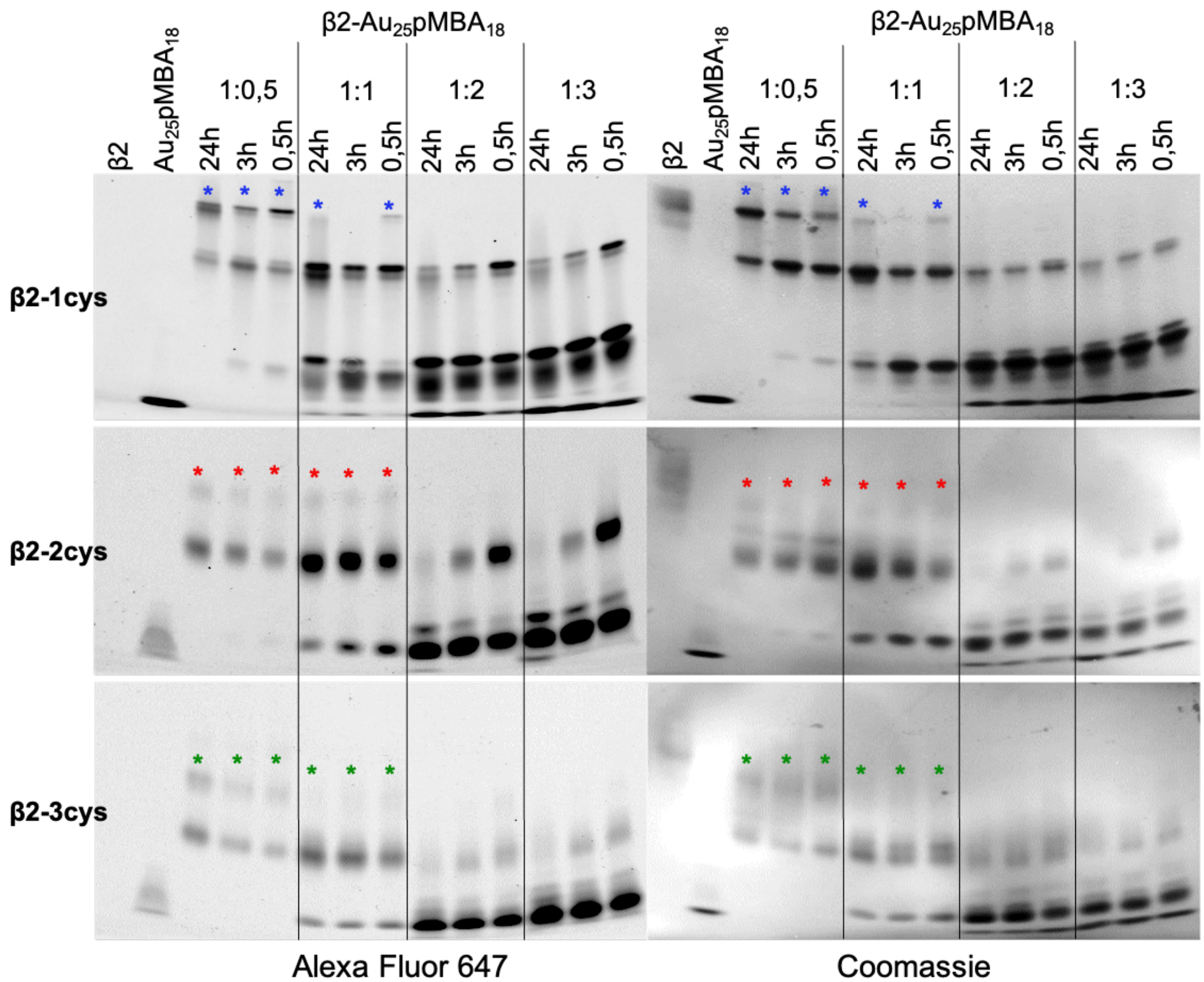

Figure S2. Population of the bioconjugates formed upon reacting $Au_{25}pMBA_{18}$ with the 3 β2 constructs (β2-1cys, β2-2cys and β2-3cys from top to bottom) depending on the reaction time (24h, 3h and 0.5h) and the protein:Au-NC stoichiometry (1:0.5, 1:1, 1:2, 1:3). Native PAGE-gels have been revealed by fluorescence emission at 667 nm (left) and Coomassie (right). * Indicate species only appearing at high protein:Au-NC stoichiometry.

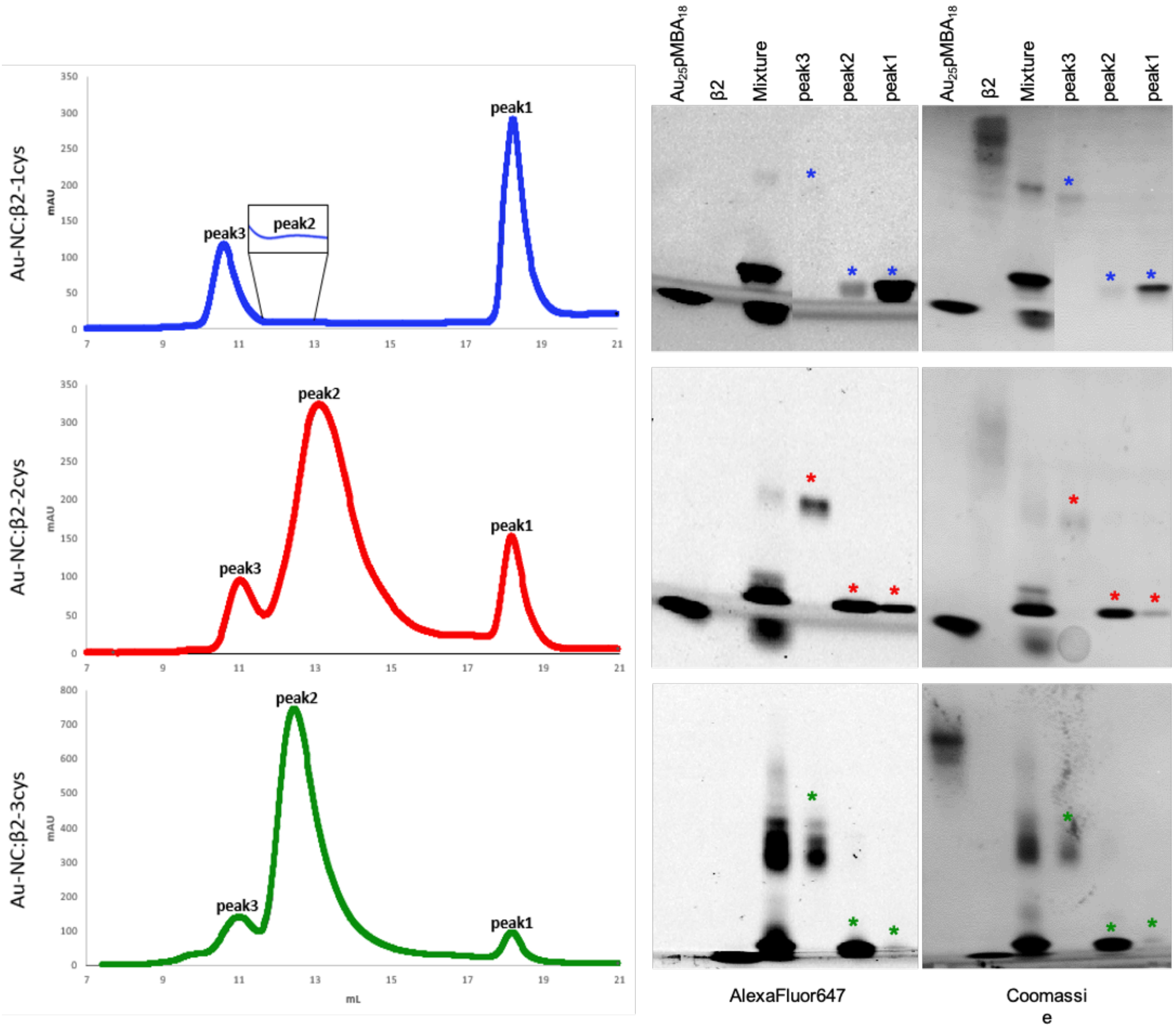

Figure S3. (Left) UV profiles of the Size-Exclusion Chromatography obtained after mixing $Au_{25}pMBA_{18}$ with β2-1cys (blue), β2-2cys (red) and β2-3cys (green). The individual fractions were collected and concentrated for the subsequent optical and structural characterization. (Right) Native PAGE-gels of the different SEC peaks revealed by fluorescence emission at 667 nm (left) and Coomassie (right). The main species for each of the SEC peaks is indicated with a *.

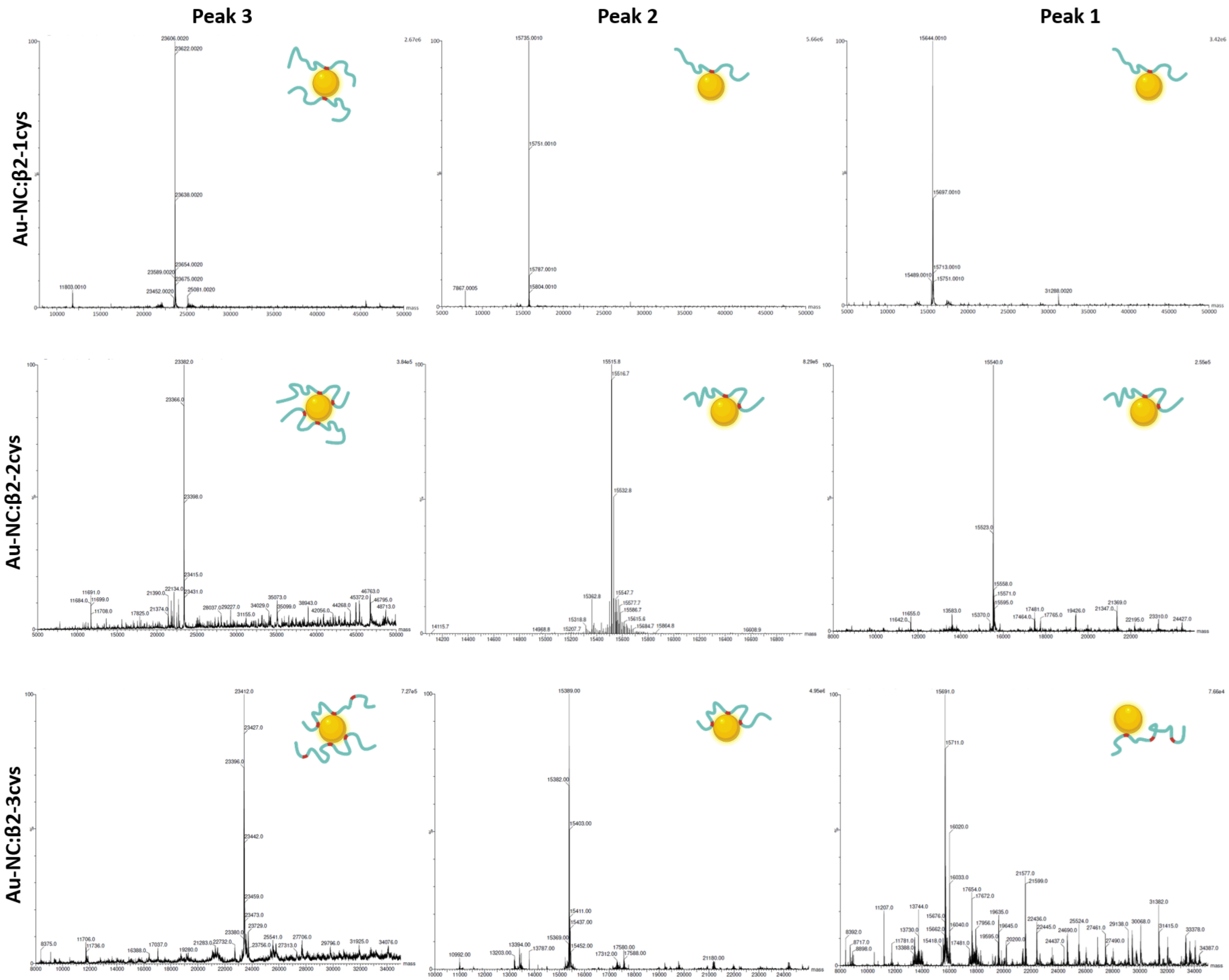

Figure S4. Electrospray mass spectra for the nine bioconjugates (three species for each of the three mixtures) isolated and characterized in this study. The molecular weight of the most intense peak is reported in table S2. Cartoons displaying the topology for each species obtained are displayed in the panels.

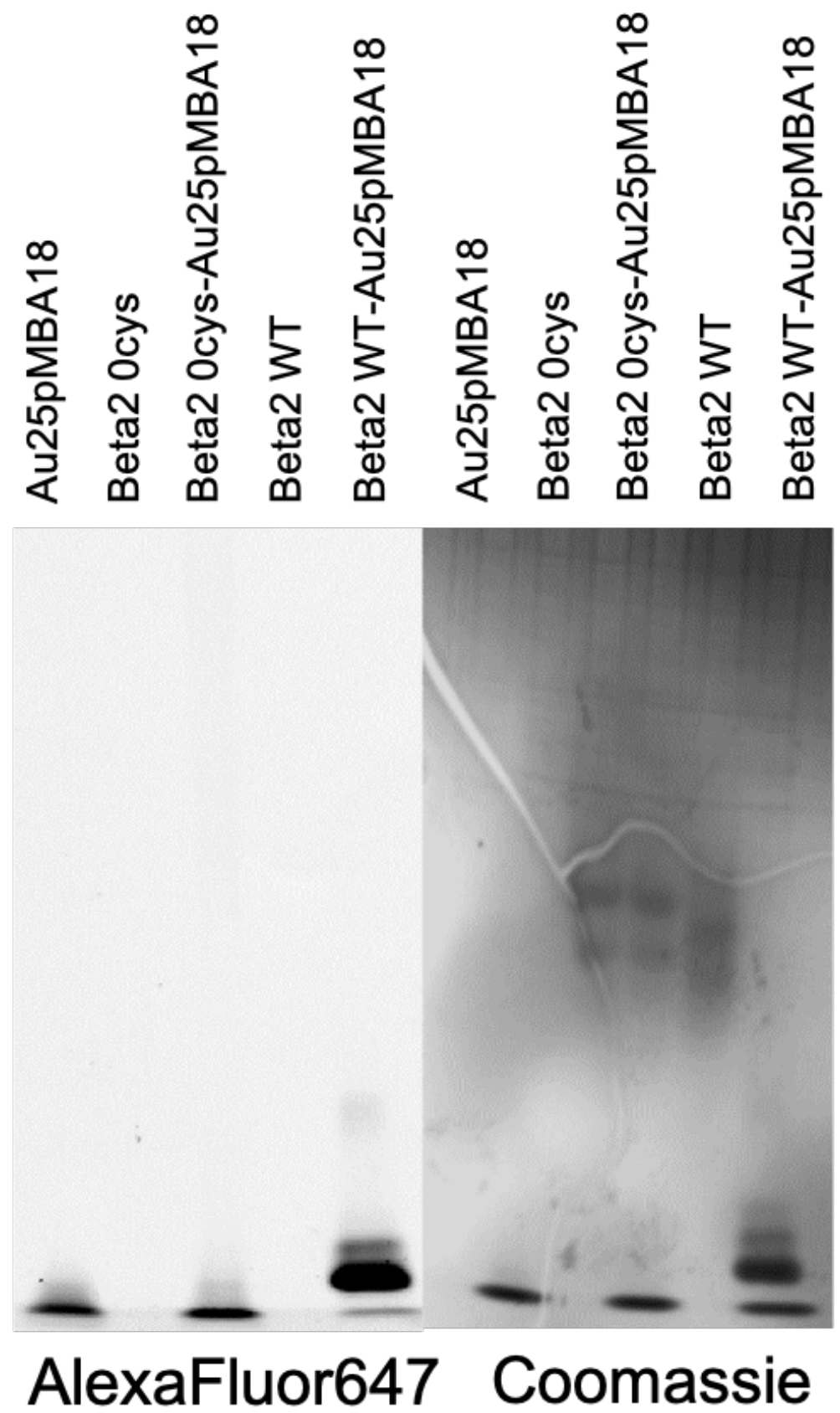

Figure S5. Native PAGE-gel revealed by fluorescence emission at 667 nm (left) and Coomassie (right) of the mixtures of the $Au_{25}pMBA_{18}$ with the β2-0cys construct. The gel shows that no complex is formed when mixing the Au-NC with a protein without free cysteines.

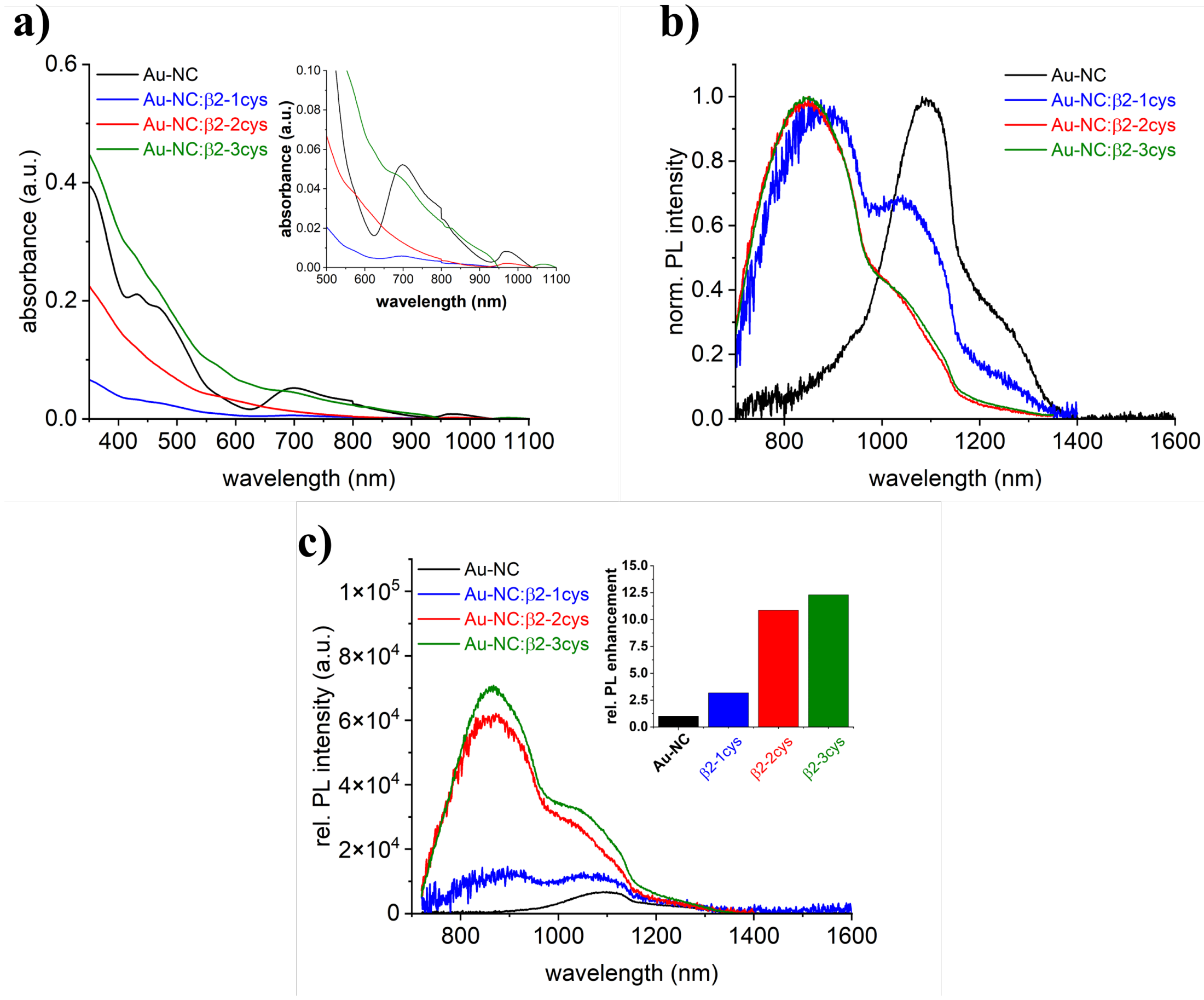

Sample	A_1	t_1 (ns)	A_2	t_2 (ns)	A_3	t_3 (ns)	$<t>_{int}$ (ns)
$Au_{25}pMBA_{18}$	0.040	6.0	0.122	44.1	0.016	259.4	**133**
Au-NC:b2 1-cys	0.171	22.5	0.173	153.9	0.083	680.0	**490**
Au-NC:b2 2-cys	0.143	35.3	0.184	250.9	0.095	908.2	**654**
Au-NC:b2 3-cys	0.125	48.1	0.172	278.7	0.110	1068.1	**811**

Figure S6. (Top) (a) Absorbance spectra of the major species of the β2 conjugates with an enlarged representation of the spectra in the range from 500 to 1100 nm as inset. **(b)** Normalized PL spectra of the major species of the β2 conjugates excited at 450 nm. **(c)** Absorbance corrected PL emission spectra of the major species of the β2 conjugates excited at 690 nm. The inset shows the calculated PL enhancement. **(Bottom)** Summary of the amplitudes (A_i) and PL lifetimes (t_i) obtained from fitting the PL decay curves using a three exponential fit and the calculated intensity weighted average PL lifetime ($<t>_{int}$).

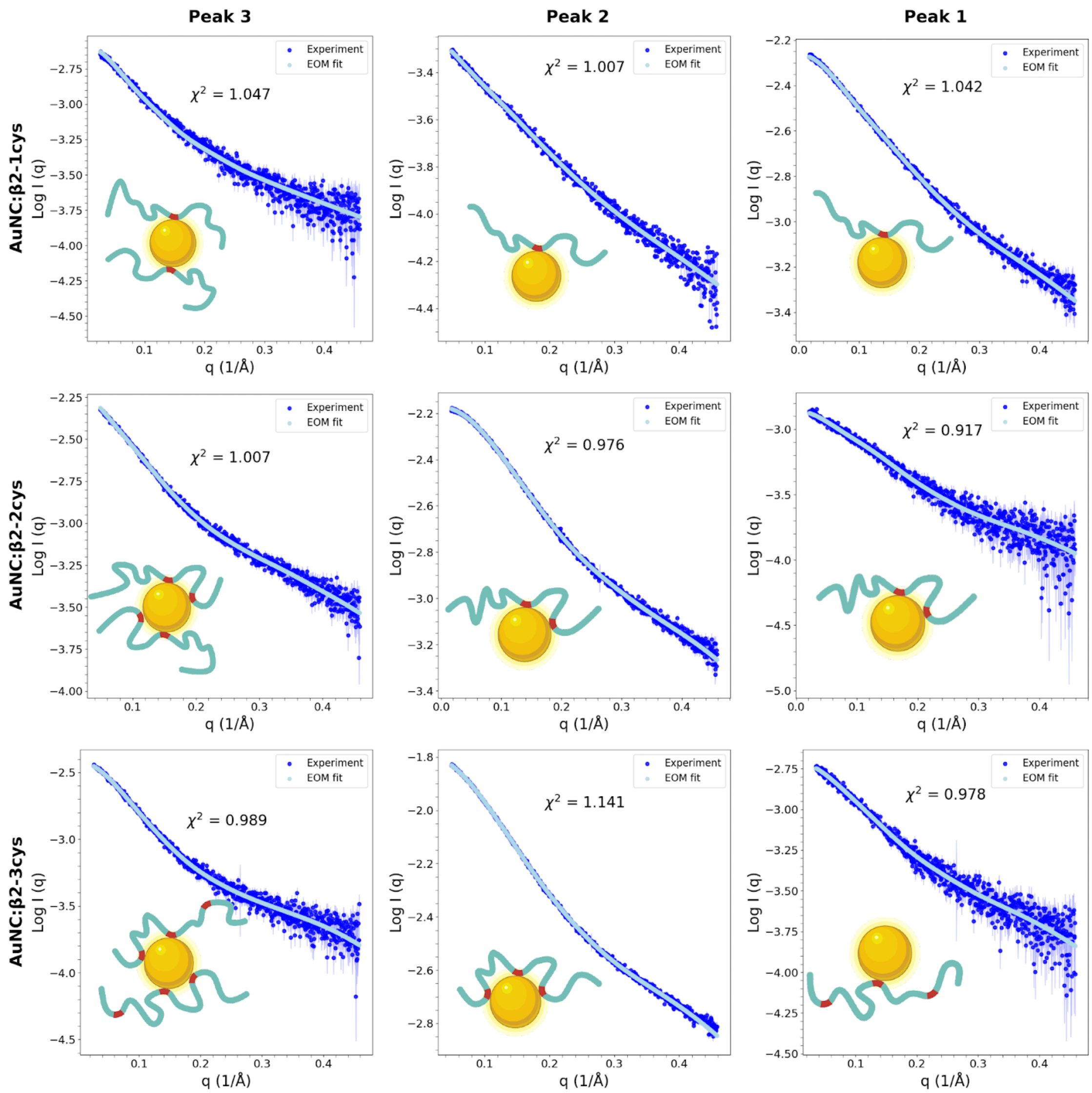

Figure S7. SAXS intensity profiles (blue dots) as a function of the momentum transfer, q, for each of the peaks purified after mixing Au-NC with the three β2 constructs. The fit using the Ensemble Optimization Method (EOM) is displayed as a solid cyan line. Cartoons displaying the topology for each species obtained by mass spectrometry and modelled for the subsequent fitting are displayed in the panels.

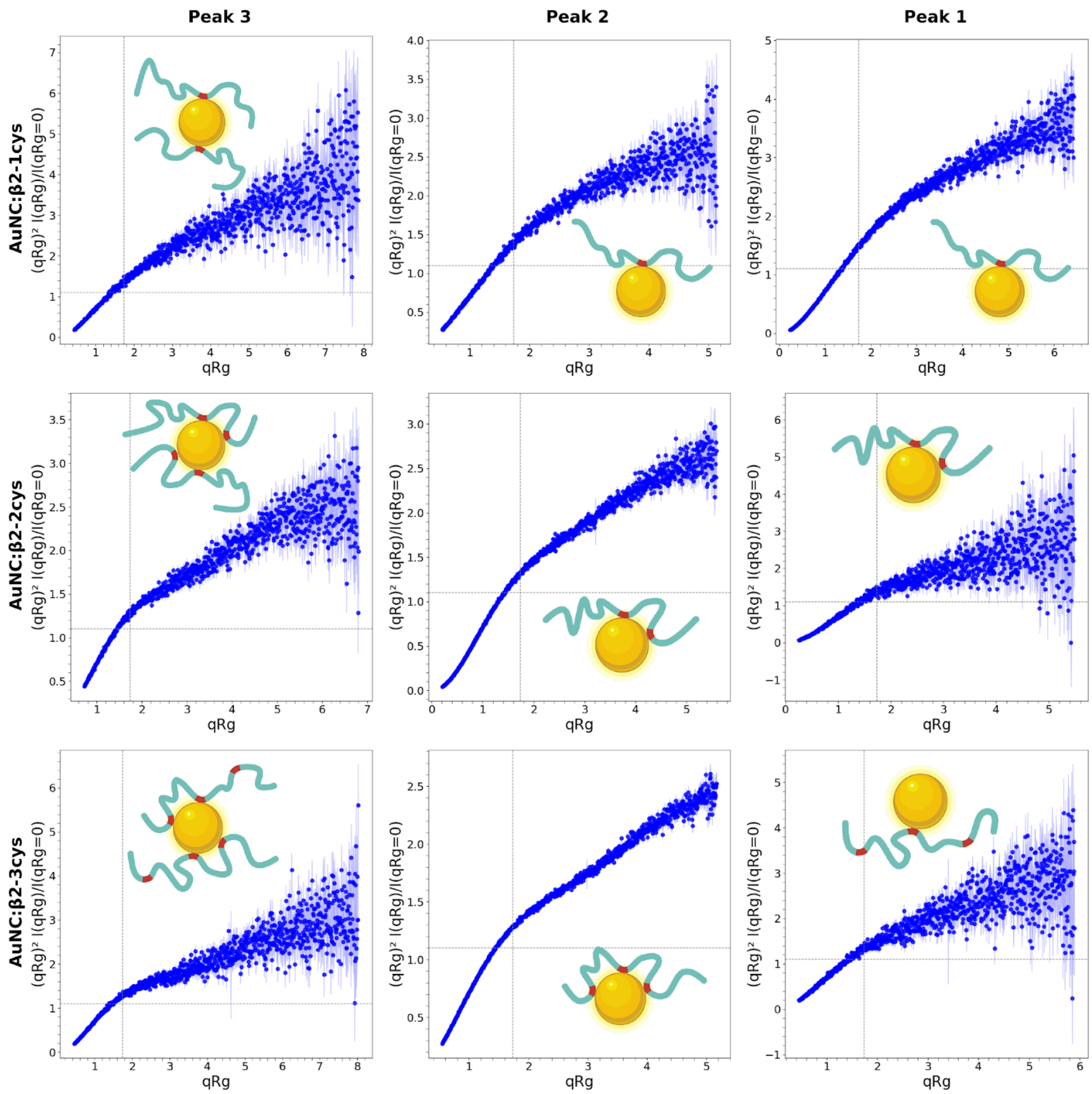

Figure S8. Dimensionless Kratky plots derived from the SAXS profiles (blue dots) for each of the peaks purified after mixing Au-NC with the three β2 constructs. Cartoons displaying the topology for each species obtained by mass spectrometry and modelled for the subsequent fitting are displayed in the panels.

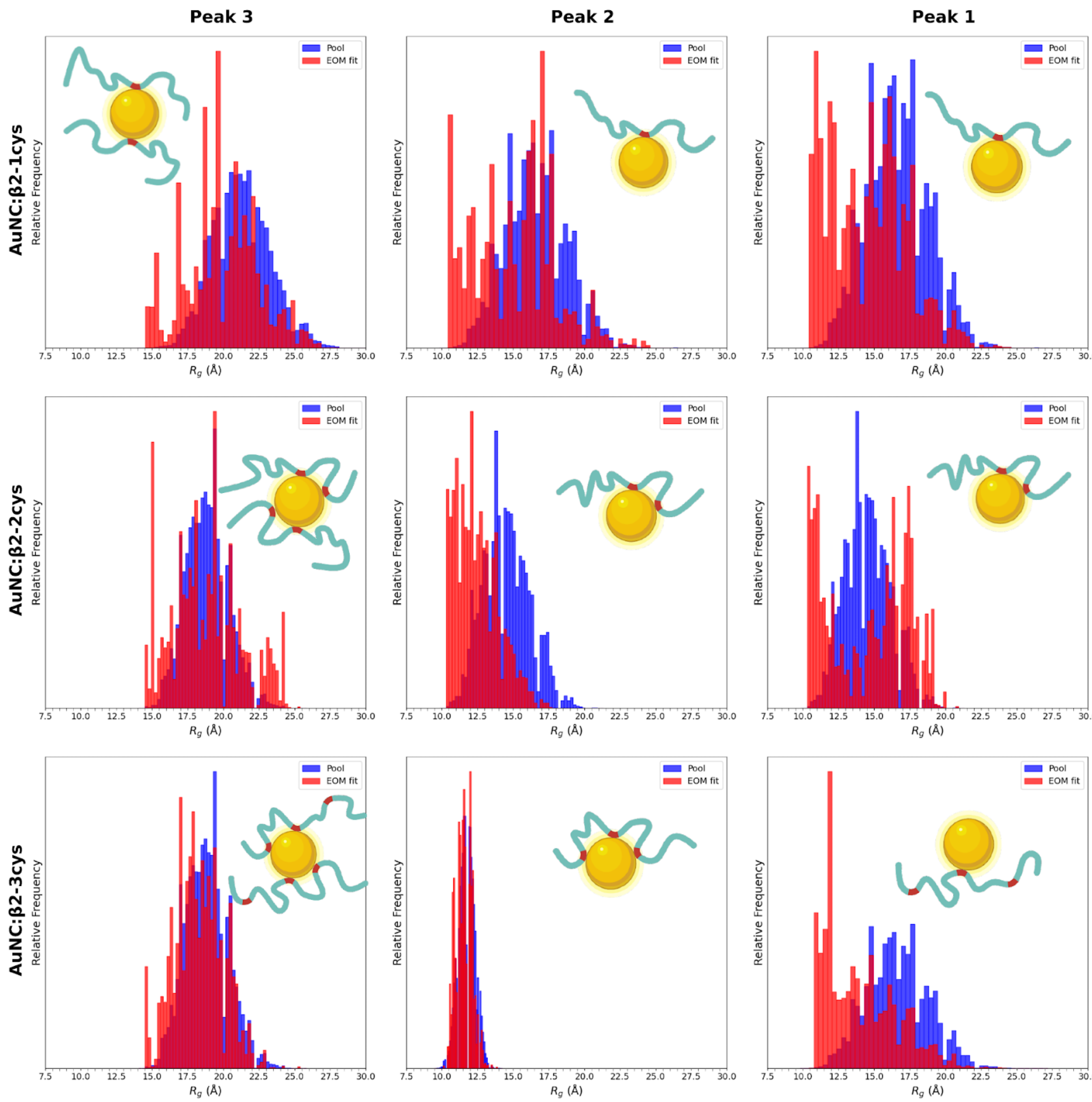

Figure S9. Comparison of the R_g distributions from the pool of conformations (blue) with these derived from the EOM analysis (red) of the SAXS curves measured for each of the peaks purified after mixing Au-NC with the three β2 constructs. Cartoons displaying the topology for each species obtained by mass spectrometry and modelled for the EOM fitting are displayed in the panels.

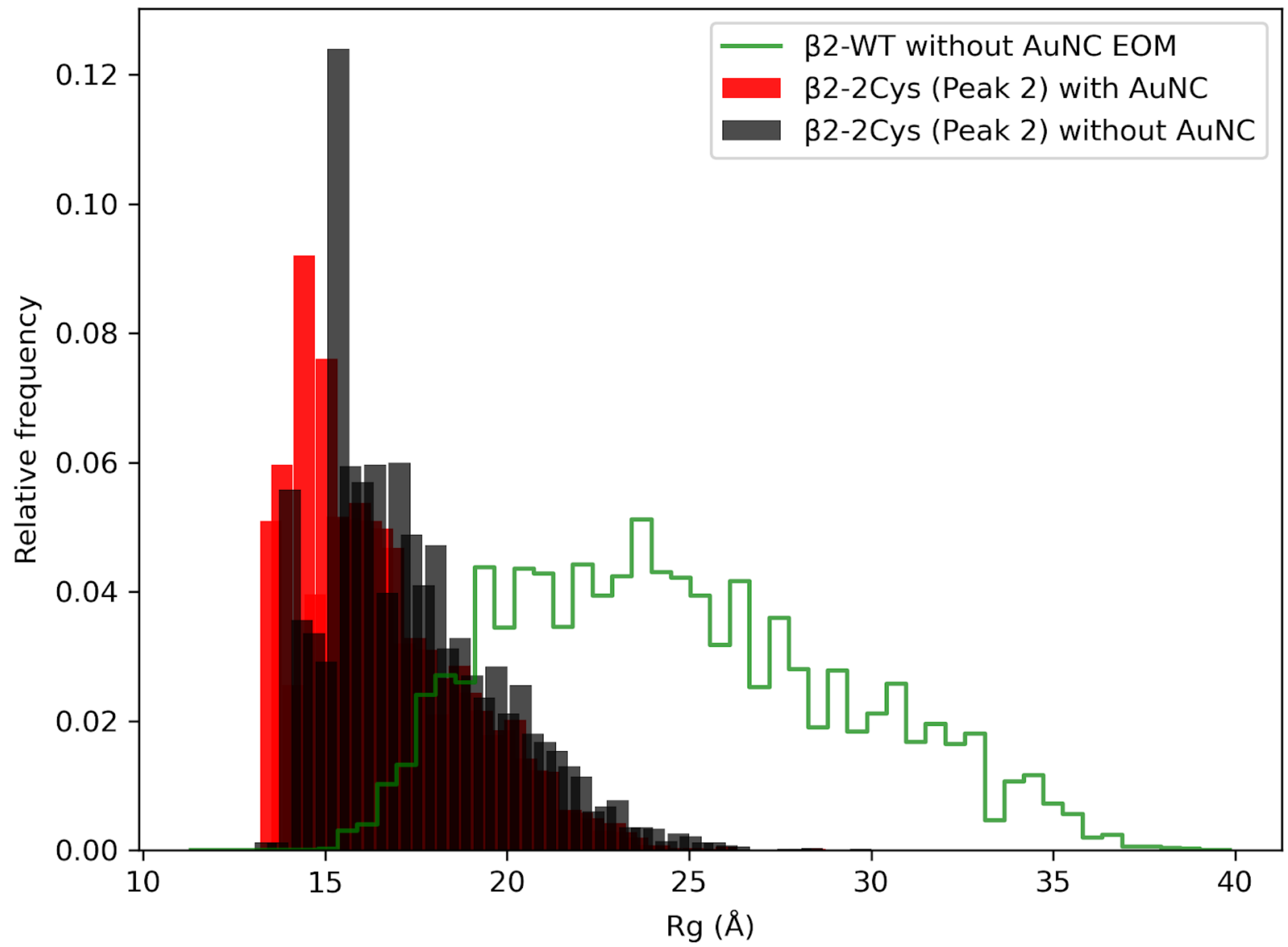

Figure S10. R_g distribution corresponding to the EOM analysis of the Peak 2 of the β2-2cys (red), compared to the R_g distribution of the same ensemble in which the Au-NC has been computationally removed before the CRYSOL calculation (gray). In green, the R_g distribution derived from the EOM analysis of the protein β2-WT. These data indicate that, in the presence of the Au-NCs, the protein displays very compact conformations, and that the compactness mainly arises from the non-covalent interactions between the protein and the nanoparticle.

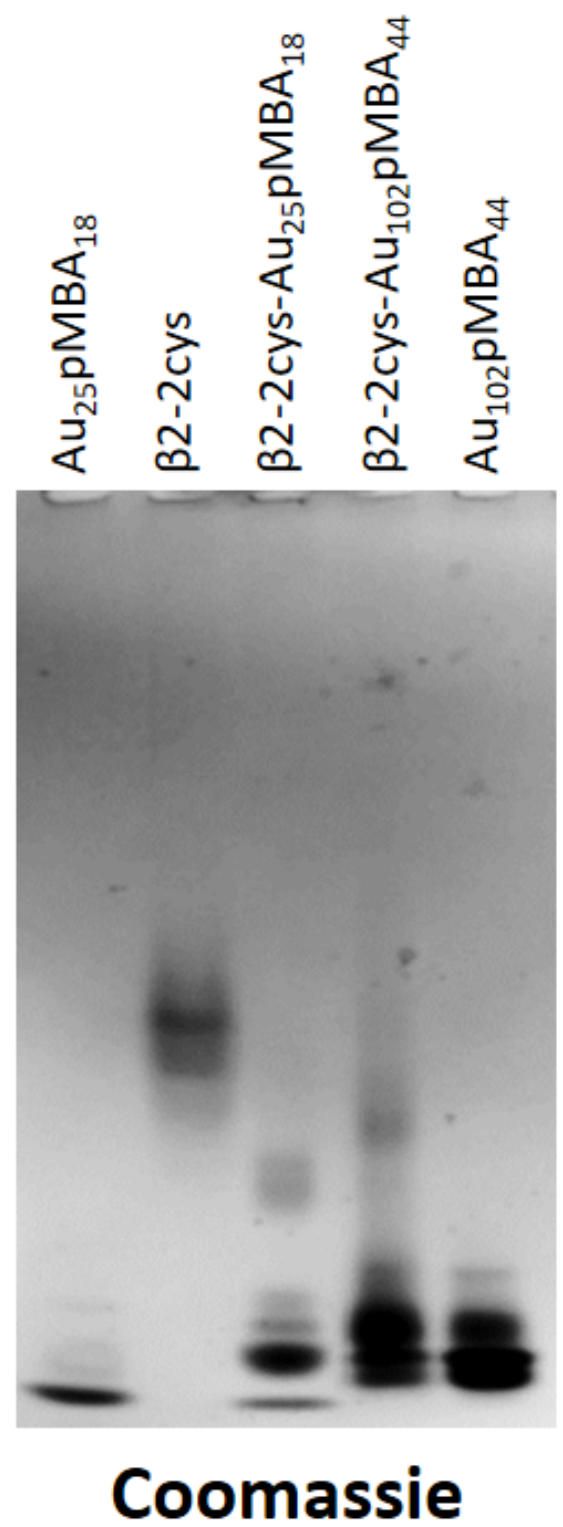

Figure S11. Generalization of the ligand exchange mechanism to other Au-NCs. Native PAGE gel of the mixtures of $Au_{25}pMBA_{18}$ and $Au_{102}pMBA_{44}$ with β2-2cys. In both cases additional bands, different from the free protein and the Au-NC, appear in the gel indicating the complexation between both molecules.

Table S1: Amino acid sequences of the 76-residue long β2 constructs used. Cysteines are highlighted in red.

Protein	Mutations	Sequence
**β2-0cys**	C39A+C67A	GAMALRRSSLKAYGNGYSSNGNTGEQSGYHVEQEKENKLLAEDLP GTEDFVGHQGTVPSDNIDSQGRNASTNDSLL
**β2-1cys**	C67A	GAMALRRSSLKAYGNGYSSNGNTGEQSGYHVEQEKENKLL**C**EDLP GTEDFVGHQGTVPSDNIDSQGRNASTNDSLL
**β2-2cys**	WT	GAMALRRSSLKAYGNGYSSNGNTGEQSGYHVEQEKENKLL**C**EDLP GTEDFVGHQGTVPSDNIDSQGRN**C**STNDSLL
**β2-3cys**	S16C	GAMALRRSSLKAYGNGY**C**SNGNTGEQSGYHVEQEKENKLL**C**EDLP GTEDFVGHQGTVPSDNIDSQGRN**C**STNDSLL

Table S2: Mass spectrometry and SAXS analyses of the purified Au-NC:β2 conjugates

	**β2-1cys**						**β2-2cys**						**β2-3cys**					
**MW_{Theo} (Da)**	8117.7						8149.7						8165.8					
	MW_{Exp} (Da)	Comp. β2/pMBA	MW_{Theo} (Da)	*SAXS Mode*	R_g (Å)	χ^2	MW_{Exp} (Da)	Comp. β2/pMBA	MW_{Theo} (Da)	*SAXS Mode*	R_g (Å)	χ^2	MW_{Exp} (Da)	Comp. β2/pMBA	MW_{Theo} (Da)	*SAXS Mode*	R_g (Å)	χ^2
**Peak 1**	15644	1/17	15643.7	BATCH	14.0 +/- 0.1	1.04	15540	1/16	15522.7	BATCH	12.0 +/- 0.1	0.92	15691.8	1/17	15691.8	SEC	12.9 +/- 0.1	0.98
**Peak 2**	15735	1/17	15643.7	BATCH	11.2 +/- 0.1	1.00	15515	1/16	15522.7	SEC	12.2 +/- 0.1	0.98	15389.0	1/15	15385.8	SEC	11.3 +/- 0.1	1.14
**Peak 3**	23606	2/16	23608.3	BATCH	17.1 +/- 0.2	1.05	23382	2/14	23366.5	BATCH	14.9 +/- 0.1	1.00	23412.0	2/14	23398.6	SEC	17.5 +/- 0.1	0.99

MW: Au 197 Da, pMBA 153 Da, $Au_{25}pMBA_{18}$ 7679 Da.

Table S3: Design of β2 constructs

Protein	Mutation	Primers	sequence 5'-3'
**β2-0cys**	C39A+C67A	Oligo MUT b2AR 1 Cys	AAAGAAAACAAACTGCTGgcCGAAGATCTGCCTGGCACC
		Oligo MUT b2AR 1 Cys C	GGTGCCAGGCAGATCTTCGgcCAGCAGTTTGTTTTCTTT
		Oligo MUT b2AR 2 Cys	GATAGCCAGGGTCGTAATgcTAGCACCAATGATAGCCTG
		Oligo MUT b2AR 2 Cys C	CAGGCTATCATTGGTGCTAgcATTACGACCCTGGCTATC
**β2-1cys**	C67A	Oligo MUT b2AR 2 Cys	GATAGCCAGGGTCGTAATgcTAGCACCAATGATAGCCTG
		Oligo MUT b2AR 2 Cys C	CAGGCTATCATTGGTGCTAgcATTACGACCCTGGCTATC
**β2-3cys**	S16C	Oligo b2AR S16C 5'	CATATGGTAATGGTTATtgcAGCAATGGCAATACCGG
		Oligo b2AR S16C 3'	CCGGTATTGCCATTGCTgcaATAACCATTACCATATG
